# Gut Microbiota Composition and Diversity in Attention-Deficit/Hyperactivity Disorder: A Systematic Review

**DOI:** 10.3390/microorganisms14061301

**Published:** 2026-06-09

**Authors:** Beatriz Rodrigues, Isabel M. Miranda, Sofia Costa de Oliveira

**Affiliations:** 1 Faculty of Medicine, The University of Porto, Alameda Professor Hernâni Monteiro, 4200-319 Porto, Portugal; beamorais02@gmail.com; 2 RISE-Health, Faculty of Medicine, The University of Porto, 4200-319 Porto, Portugal; imiranda@med.up.pt

**Keywords:** ADHD, gut microbiota, gut-brain axis, microbiome

## Abstract

Attention-deficit/hyperactivity disorder (ADHD) is a common neurodevelopmental condition. Growing evidence suggests that the gut–brain axis may contribute to its pathophysiology. However, findings regarding gut microbiota alterations in ADHD remain inconsistent. This systematic review aimed to synthesize the current evidence on the gut microbiota composition and microbial diversity in individuals with ADHD. A systematic search of PubMed, Scopus, and Web of Science was conducted up to 31 December 2025 following PRISMA guidelines, yielding 562 studies. Twenty-three studies published between 2015 and 2025 were included. Most studies reported no significant differences in alpha-diversity in ADHD and control groups. More consistently, beta-diversity analysis reported significant differences in microbial composition between ADHD and control groups. ADHD was often associated with a reduced abundance of *Alistipes* and butyrate producers such as *Faecalibacterium* and increased abundance of *Roseburia* and *Agathobacter*. Some longitudinal studies suggested that distinct early-life microbial patterns may precede the ADHD diagnosis. ADHD appears to be associated with alterations in the gut microbiota, particularly in taxa involved in short-chain fatty acid production and immune regulation. However, findings remain inconsistent due to methodological heterogeneity and potential confounding factors. Future research should prioritize longitudinal multi-omics approaches to clarify causal mechanisms and refine microbiota-targeted interventions.

## 1. Introduction

Attention-deficit/hyperactivity disorder (ADHD) represents a neurodevelopmental disorder, and its diagnosis has increased significantly over recent years [1,2,3]. This condition is characterized by symptoms of inattention, hyperactivity, and/or impulsivity [4]. Approximately 8% of children and adolescents across the world are diagnosed with ADHD, and in the majority of them, the symptoms persist into adulthood [5]. In fact, an umbrella review estimates a 3.1% global prevalence for adult ADHD, higher than other well-known mental health conditions such as bipolar disorder and anxiety/panic disorders [6]. This condition has a significant impact on the daily life of those affected. This population has a reduced quality of life, lower educational attainment and occupational functioning, difficulties in relationships, and a greater risk of criminality and other mental health disorders [7]. A systematic review from 2021 estimated a total cost per person ranging from 831.38 to 20,538 US dollars and national costs ranging from 356 million to 20.27 billion US dollars [8]. For that reason, this disorder represents a burden not only for affected individuals and their surroundings, but also for society and healthcare systems [9].

Over the last decade, the literature on the gut microbiota has grown dramatically, especially in areas studying its connection to neurodevelopmental and psychiatric disorders. This concept is known as the gut–brain axis, a communication network between the central nervous system (CNS) and the gastrointestinal tract. It is a complex bidirectional system that occurs through neural, endocrine, and immune pathways [10]. Certain intestinal bacteria can secrete neurotransmitters such as gamma-aminobutyric acid (GABA), dopamine, and serotonin, which have been implicated in ADHD pathophysiology [11]. The modulation of these neurotransmitters is mediated by specific peptides and metabolites, also released by the gut microbiota. In addition, the vagus nerve may also play an important role, as children with ADHD are found to have reduced vagus nerve activity and parasympathetic tone [12]. Lastly, gut dysbiosis can lead to increased gut permeability, resulting in systemic inflammation. This may help explain why elevated markers of inflammation have been reported in ADHD patients [13]. All of these represent possible ways the gut microbiota could interfere with brain mechanisms relevant to ADHD.

Findings from animal studies show that mice, after being transplanted with fecal microbiota from individuals with and without ADHD, have an altered brain structure and function and even changes in their behavior [14]. Regarding human research, differences in gut microbiota composition have been documented between those with ADHD and those without [15]. More recent studies suggest that supplementation with probiotics may improve ADHD symptomatology, reducing hyperactivity and increasing academic performance [16]. However, findings have been inconsistent across studies, and recent systematic reviews highlight several limitations, including the poor control of confounding factors (e.g., diet, medication, or age) [17], small sample sizes [18,19], and heterogeneous methodologies [17].

Considering all of this, clarifying the relationship between gut microbiota and ADHD is crucial for a better understanding of this pathology and opening new doors to microbiota-targeted interventions such as probiotics, prebiotics, and dietary modulation, with the goal of improving ADHD symptoms and the quality of life of patients.

The purpose of this systematic review was to evaluate and synthesize the existing evidence on the gut microbiota composition, microbial diversity, and their potential clinical implications in ADHD. Specifically, this review aimed to synthesize evidence from studies evaluating the gut microbiota composition and microbial diversity in individuals with ADHD, including comparisons with healthy control groups when available, and to explore how these microbiota features may relate to ADHD symptomatology or treatment responses.

## 2. Materials and Methods

The systematic review was carried out in strict accordance with the Cochrane Handbook for Systematic Reviews of Interventions [20] and the Preferred Reporting Items for Systematic Reviews and Meta-Analyses (PRISMA) checklist [21] was followed (Supplementary Materials Table S1).

### 2.1. Search Strategy

A systematic search strategy was performed from database inception until 31 December 2025 in three databases: PubMed, Scopus, and Web of Science. A combination of Medical Subject Headings (MeSH) terms and keywords was used as follows: (ADHD OR “attention-deficit” OR hyperactivity OR “hyperactivity disorder”) AND (microbiota* OR microbiome*) AND (child* OR children*). MeSH terms and keywords were selected to capture studies related to ADHD, pediatric populations, and the gut microbiota composition and diversity. No language restrictions were applied during the search process. However, all studies ultimately included in the final analysis were published in English.

### 2.2. Eligibility Criteria

The search included all original studies published up to 31 December 2025, excluding reviews, systematic reviews, and editorials. Articles were included based on the following criteria: (i) studies performed on humans under 18 years old with a diagnosis of ADHD; (ii) studies addressing the gut or intestinal microbiota and/or microbiome analysis; and (iii) studies including a control group. Comparisons between ADHD and non-ADHD populations were derived from the control groups included within eligible studies.

### 2.3. Study Selection and Data Extraction

**Figure 1:**
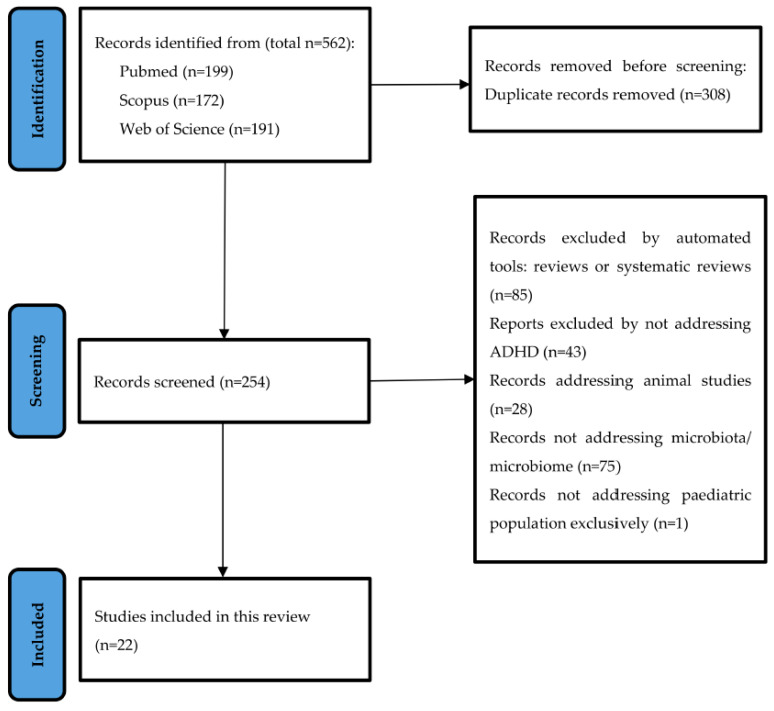
PRISMA flowchart representing the studies identified during the systematic search and analysis.

A total of 562 references were obtained in the three databases. The extracted studies were uploaded to EndNote 20.6 and the Rayyan software [22] for duplicate removal and further selection. In a blinded, standardized manner, screening of the title, abstract, and full text was performed by two independent reviewers (BR and SCO) based on the inclusion criteria. Disagreements were resolved via consensus. The PRISMA flowchart (Figure 1) summarizes the literature search and screening strategies.

A protocol was established to uniformly and consistently synthesize the data collected from the selected studies. Data were organized according to the study characteristics, microbiota assessment methodology, microbial taxonomy, and diversity. No meta-analysis was performed due to the heterogeneity of the studies. This heterogeneity involved multiple dimensions, including clinical variability (age ranges, medication exposure, comorbidities), methodological differences (study design, sequencing platforms, DNA extraction protocols), statistical heterogeneity (different alpha- and beta-diversity metrics), and outcome heterogeneity related to taxonomic reporting. Given these differences, quantitative pooling could generate misleading summary estimates and reduce the interpretability of findings.

### 2.4. Risk of Bias (RoB) Assessment

Risk of bias was assessed at the study level using design-appropriate tools, as the included gut microbiota–ADHD literature included randomized trials (Cochrane RoB 2), non-randomized interventions (ROBINS-I), observational and case-control studies (Newcastle-Ottawa Scale), cross-sectional studies (Joanna Briggs Institute Critical Appraisal Checklist), and Mendelian randomization (ROB-MR). Two reviewers (SCO and IMM) independently completed the assessments using full texts and resolved disagreements through consensus. 

## 3. Results

### 3.1. Study Characteristics

This review included a total of 22 studies, published between 2015 and 2025 [23,24,25,26,27,28,29,30,31,32,33,34,35,36,37,38,39,40,41,42,43,44]. The characteristics of the included studies are summarized in Table 1. The included study designs comprised 12 cross-sectional studies, 6 randomized controlled trials, 3 prospective cohort studies, and 1 two-sample Mendelian randomization (MR) study.

Studies were conducted across Europe, Asia (including China, Japan, South Korea, Thailand, and Taiwan), Oceania (New Zealand), and North America. Asian countries were the most frequently represented, particularly China and Taiwan, accounting for the largest proportion of the included research [25,28,29,30,31,32,35,36,38,39,40,41,43]. European countries also contributed significantly (Finland, Germany, Denmark, Sweden, United Kingdom, Spain, and Italy) [23,25,32,36,43,44]. Fewer studies were conducted in North America (USA and USA/Canada) [33,41] and Oceania (New Zealand) [26]. Overall, Asia constituted the most common geographical setting among the included studies.

Study populations varied widely in size, ranging from 17 participants [26] to 16,640 individuals in a national cohort study [36]. Most cross-sectional microbiome analyses included the 30 to 110 participant range, while intervention trials were typically smaller (*n* = 17–76).

Participants were predominantly children and adolescents aged 6–16 years. However, some cohorts followed participants from birth into adolescence [23,33,36] Most samples were male-predominant, with male representation frequently exceeding 60%, consistent with ADHD epidemiology.

#### 3.1.1. ADHD Diagnosis

The ADHD diagnosis was primarily based on Diagnostic and Statistical Manual of Mental Disorders (DSM-IV, DSM-IV-TR, or DSM-5) criteria, commonly assessed through structured clinical interviews such as the Kiddie Schedule for Affective Disorders and Schizophrenia (K-SADS) and its various versions [24,25,27,28,31,39] or through validated rating scales such as the ADHD Rating Scale (ADHD-RS), Conners, Swanson, Nolan, and Pelham Questionnaire IV (SNAP-IV), and Korean ADHD Rating Scale (K-ARS) [27,30,37]. A smaller subset of studies relied on International Classification of Diseases (ICD-10) criteria [23,32,35,36]. Differences in diagnostic criteria across studies may contribute to increased heterogeneity in findings.

#### 3.1.2. Medication Status

Medication status varied across studies. While several cross-sectional studies excluded medicated participants [24,28,30,31,34,39], others included both medicated and unmedicated individuals [32,37,42]. Some studies required medication washout periods prior to sampling [25].

In intervention trials, participants were either medication-free [41,44], on stable methylphenidate treatment [40], or receiving standard ADHD pharmacotherapy alongside dietary intervention [38]. Among the pharmacological treatments reported, the most commonly used medication was a psychostimulant (methylphenidate). Medication use represents a potential confounder since psychostimulants may influence the composition of the gut microbiota.

#### 3.1.3. Dietary Assessment

Dietary intake was variably assessed across studies. Some studies excluded participants following a vegetarian diet [24,27,28,31,34,40], whereas others recorded dietary intake by using Food Frequency Questionnaires (FFQ) or dietary diaries [25,26,29,37,42,43]. One study reported no significant differences between the ADHD and control groups [42], whereas others identified differences in dietary patterns between groups [28,29].

#### 3.1.4. Probiotic/Prebiotic and Antibiotic Use

Antibiotic and probiotic use were frequently identified as potential confounding factors. For this reason, most studies excluded participants with recent antibiotic or probiotic exposure within 2 weeks to 3 months prior to sample collection [24,26,27,28,29,30,31,32,34,37,39,40,42,43], although exclusion criteria varied considerably between studies. Some studies also recorded antibiotic use without excluding participants [23,41].

Early-life antibiotic exposure was examined in one large longitudinal cohort study and it was reported that children experiencing three or more antibiotic-requiring infections had a higher risk of developing ADHD. Those with six or more infections requiring antibiotic treatment showed an even higher risk [36].

Notably, several studies did not systematically assess or report participants’ medication status, dietary patterns, or probiotic and antibiotic use.

#### 3.1.5. Interventions

The intervention studies comprised several microbiota-targeted and nutritional approaches. These included perinatal probiotic supplementation with *Lactobacillus rhamnosus* GG [23], add-on supplementation with *Bifidobacterium bifidum* (Bf-688) [40], multistrain probiotic or synbiotic supplementation [43,44], micronutrient supplementation [26,41], and a dietary intervention by introducing a low-lectin diet [38].

Most intervention trials lasted between 6 and 16 weeks, with one long-term perinatal follow-up study extending up to 13 years [23].

### 3.2. Microbiota/Microbiome Methodology

In all studies, fecal samples were stored at −80 °C until extraction (Table 2). Deoxyribonucleic acid (DNA) extraction methods varied considerably across studies. Frequently used kits included the following: QIAamp DNA Stool Mini Kit or PowerFecal kits (Qiagen), DNeasy Blood and Tissue Kit, FastDNA Spin Kit for Soil, PowerSoil Pro extraction kit. Automated extraction systems were used in some studies [23,32]. The characterization of gut microbial communities was assessed via 16S rRNA gene sequencing using Illumina MiSeq platforms, being used in approximately 18 studies. Targeted regions varied across studies and included V1–V2, V3–V4, and V4 regions.

Two studies used shotgun metagenomic sequencing [27,39], enabling species-level taxonomic resolution and functional profiling. One Mendelian randomization study used GWAS (genome-wide association studies) summary statistics rather than direct microbiome sequencing [35]. Findings from shotgun metagenomics should be interpreted separately from those from 16S rRNA studies, as these approaches differ substantially in taxonomic resolution and functional inference capacity.

Fungal analyses (mycobiome) were conducted in one cohort study using the internal transcribed spacer 2 (ITS2) sequencing region [34].

Considerable heterogeneity was observed in the reference databases used for taxonomic classification, and these included SILVA, GreenGenes, Ribosomal Database Project (RDP), MetaPhlAn2, EzBioCloud, and the Unified Human Gastrointestinal Genome (UHGG) database. These methodological differences may influence the microbial composition and constitute an important source of heterogeneity across studies.

### 3.3. Diversity Analysis

Alpha diversity, beta diversity, and taxonomic abundance represent distinct biological constructs. Alpha diversity reflects microbial richness and evenness within a sample, while beta diversity evaluates differences in microbial compositions between groups. Taxonomic abundance refers to the relative presence of specific microbial taxa. Therefore, these outcomes were analyzed and interpreted separately throughout this review. Also, the term “significant” used throughout this review refers to statistical significance as defined within the individual included studies.

#### 3.3.1. Alpha-Diversity

Microbiota diversity and composition in the ADHD population are described in Table 3. Across the 22 included studies, results regarding microbial richness and evenness (alpha-diversity) were largely inconsistent. Most cross-sectional analyses reported no significant differences between individuals with ADHD and controls across several diversity indices, such as Shannon, Chao1, Simpson, Abundance-based Coverage Estimator (ACE), and Faith’s phylogenetic diversity [24,27,30,31,34,37].

When significant differences were detected, the contributing metrics varied across studies. Reductions limited to the Shannon index were reported in one cohort [25], while another study showed decreases in both Shannon and Simpson indices [39]. In other studies, differences were driven primarily by reduced richness measures, including amplicon sequence variant (ASV) richness and Faith’s phylogenetic diversity [32], or described as an overall decrease in alpha-diversity without specifying the contributing indices [29]. Conversely, some studies reported a higher alpha-diversity in ADHD, including higher Chao1 and Shannon with lower Simpson [28], as well as higher Faith’s phylogenetic diversity [42]. Longitudinal studies suggested that associations may be age-dependent. Higher Faith’s phylogenetic diversity at 6 months was observed in children who later developed ADHD [33].

Intervention studies yielded inconsistent results. Micronutrient supplementation increased evenness-related metrics [41]. Probiotic trials reported significant changes in Shannon and Chao1 in both intervention and placebo groups in one study [40], while another reported no significant changes [43]. A low-lectin diet did not significantly modify alpha-diversity [38].

Additionally, psychostimulant treatment was associated with lower alpha-diversity compared with unmedicated individuals with ADHD [42].

#### 3.3.2. Beta-Diversity

Beta-diversity findings remained heterogeneous, although between-group differences were reported more consistently than for alpha-diversity. Several studies reported significant differences in the overall microbial community composition between individuals with ADHD and controls using UniFrac, Bray–Curtis, or Jaccard distance metrics [25,29,30,32,34,39,42]. In contrast, other studies did not observe clear group separation, often attributing this finding to high interindividual variability in microbial composition [24,27].

In longitudinal analyses, microbial composition at 6 months of age differed between children who later developed ADHD and neurotypical controls [33].

Among intervention studies, micronutrient supplementation and probiotic treatment significantly altered beta-diversity compared with the placebo in some trials [40,41], whereas others reported no pre–post differences [26,43]. Additionally, psychostimulant medication was associated with distinct microbial community profiles compared with unmedicated individuals with ADHD [42].

### 3.4. Taxonomic Composition

Table 4 summarizes the results achieved regarding the taxonomic changes across studies. ADHD was most frequently associated with decreased *Firmicutes* [42] and increased *Proteobacteria* (Table 3 and Table 4) [30,42]. Findings for *Actinobacteria* were inconsistent across studies [42]. Additional findings included increased *Fusobacteria* [28] and decreased *Verrucomicrobiota*, *Desulfobacterota*, and *Bacteroidota* [42]. *Prevotellaceae* was the most consistently reduced family in ADHD [25,42], whereas other families, including *Rikenellaceae*, *Porphyromonadaceae*, and *Ruminococcaceae*, showed discordant results across studies [25,27,30,31].

Several recurrent patterns were observed at the genus level. *Faecalibacterium* [24,27,29] and *Alistipes* [30,39] were most frequently reduced. By contrast, *Roseburia* [30,31], and *Agathobacter* [30,31] were commonly increased. Several genera (including *Bifidobacterium*, *Odoribacter*, *Parabacteroides*, *Prevotella*, and *Lactobacillus*) showed inconsistent trends. Many additional taxa were reported in single studies only, precluding a meaningful comparison across studies.

*Bacteroides uniformis* was the most consistently increased species in ADHD [28,31], whereas other species showed conflicting results across studies.

#### 3.4.1. Longitudinal Studies: Microbial Trajectories

Early-life microbiota differed between children who later developed ADHD and controls. At 1 month of age, children who later developed ADHD exhibited a lower abundance of *Dorea*, *Pseudoramibacter*, *Eubacterium*, *Lachnospiraceae*, *Prevotella*, and *Veillonella* and a higher abundance of *Megasphaera*, *Clostridiaceae*, *Lactococcus*, *Bacteroides*, *Streptococcus*, *Akkermansia muciniphila*, and *Collinsella aerofaciens* [33]. At 6 months, a lower abundance of 51 bacterial operational taxonomic units (OTUs), including *Enterococcus*, *Ruminococcus*, and multiple *Lactobacillales*, and a higher abundance of *Dorea* and *Blautia* were reported [33]. Another longitudinal study reported higher levels of *Megamonas funiformis* and *Veillonella parvula* and lower *Bifidobacterium*, *Coprococcus*, *Alistipes*, *Roseburia*, *Akkermansia*, and *Phascolarctobacterium* in ADHD [36].

#### 3.4.2. Effects of Interventions

Early-life supplementation with *Lactobacillus rhamnosus* GG was associated with a lower incidence of later neuropsychiatric disorders (17.1% in the placebo group vs. 0% in the probiotic; *p* = 0.008) [23]. In established ADHD, probiotics were associated with changes in the microbial composition, increasing *Odoribacter* and decreasing *Escherichia–Shigella* [43]. Micronutrient supplementation reduced *Actinobacteria* abundance compared with the placebo [26,41] and modestly increased *Proteobacteria* in the intervention group only [26]. Furthermore, *Bifidobacterium bifidum* (Bf-688) supplementation was found to increase the *Firmicutes/Bacteroidetes* ratio [40]. Regarding dietary intervention, a low-lectin diet combined with standard treatment resulted in post-treatment dominance of *Lactobacillaceae*, *Pasteurellaceae*, and *Pasteurellales* [38].

Psychostimulant medication significantly influenced the microbial landscape, being associated with a lower abundance of nine distinct genera, while specifically increasing the abundance of *Anaerostipes* [42].

### 3.5. Mycobiome

Only two of the 23 included studies investigated the mycobiome, both assessing fungal taxonomic composition [33,34]. At the phylum level, ADHD was associated with increased *Ascomycota* and decreased *Basidiomycota* [34]. At lower taxonomic levels, *Candida* was enriched in individuals with ADHD, with increased levels of *C. albicans* and *C. tropicalis* [34]. In early-life cohorts, several fungal OTUs were reduced at 1 month in children who later developed ADHD. At 6 months, *Candida* and *Saccharomyces* were reduced, whereas *Trichosporon* was increased [33].

### 3.6. Causal Inference Through Mendelian Randomization

Genomic proxy markers identified specific genera associated with an increased ADHD risk, including the *Eubacterium hallii* group, *Ruminococcaceae UCG013*, and the *Ruminococcus torques* group. Conversely, several taxa were identified as potentially protective, such as *Butyricicoccus*, *Roseburia*, *Desulfovibrio*, *Romboutsia*, *Oxalobacteraceae*, and *Peptostreptococcaceae* [35]. Reverse causality was suggested for *Roseburia* [35].

### 3.7. Microbiota Correlation with Symptom Severity and Clinical Improvement

Several taxa were associated with symptom severity. *Faecalibacterium* was negatively correlated with total and hyperactivity scores [24]. *Ruminococcus gnavus*, *Agathobacter*, and *Acidaminococcus* were associated with behavioral and emotional symptoms after an adjustment for confounders [30]. *Sutterella stercoricanis* and *Bacteroides ovatus* were positively correlated with hyperactivity scores [28]. Higher *Bifidobacterium* abundance was associated with lower ADHD-IV-RS scores [26]. No associations with Child Behaviour Checklist (CBCL) scales were observed in one study [25].

Clinical improvement following micronutrients was associated with increased *Oscillospiraceae* and *Rikenellaceae* [41] and correlated with reduced *Actinobacteria* and improved Child Global Assessment Scale (C-GAS) scores [26]. Bf-688 improved Continuous Performance Test (CPT) performance [40]. A low-lectin diet improved multiple clinical ratings [38]. A multistrain synbiotic produced small parent-reported improvements not directly linked to microbiota changes [44].

### 3.8. Risk of Bias–Quality Assessment

Across the 22 included studies (Supplementary Material Tables S2 and S3), the overall risk of bias was most frequently rated as some concerns (n = 6) or moderate (n = 6), with a substantial proportion of moderate–high (n = 4), high (n = 4) or serious (n = 1) concerns. Among the seven randomized trials assessed with RoB 2, six were judged to have some concerns and one was judged to be at high risk, largely driven by missing outcome data and a long follow-up. For the ten case-control studies assessed with the Newcastle-Ottawa Scale, risk of bias was moderate to moderate–high, with the main limitations relating to the selection of controls and the incomplete control of confounding (particularly diet, antibiotic/probiotic exposure, comorbidities, and ADHD medication). Cross-sectional studies according to the JBI checklist ranged from moderate to high risk, mainly due to residual confounding and small samples. Cohort studies generally showed a lower risk, reflecting stronger temporality but remaining vulnerable to attrition and residual confounding. The single non-randomized dietary intervention study assessed with ROBINS-I was judged to be at a serious risk of bias, primarily due to confounding and deviations from the intended interventions, while the Mendelian randomization study assessed with ROB-MR was rated as having some concerns, reflecting potential instrument weakness and horizontal pleiotropy despite sensitivity analyses.

## 4. Discussion

This systematic review synthesized evidence from 22 studies exploring the relationship between the gut microbiota composition and ADHD.

Overall, the findings suggest a potential association between this neurodevelopmental disorder and alterations in gut microbial communities. However, the direction and magnitude of these differences were highly heterogeneous and seem to depend on several factors, including age, medication status, dietary patterns, and methodological differences in microbiome analysis. The interpretation of microbiota findings should consider that medication exposure was inconsistently reported across studies, and several studies included a mixed population of medicated and non-medicated participants without separate subgroup analysis. Consequently, the potential confounding effects of psychostimulant treatment on the gut microbiota composition could not be fully disentangled.

### 4.1. Diversity Findings

Alpha-diversity findings were inconsistent across studies, with the majority reporting no significant differences between ADHD and controls, whereas a minority described either reduced or increased diversity. These discrepancies were driven by distinct diversity indices across studies, potentially implying that richness and evenness may be differentially affected in ADHD. This variability likely reflects methodological heterogeneity but may also indicate that global diversity is not the most sensitive marker of dysbiosis related to ADHD. Instead, this disorder may be better characterized by changes in specific microbial taxa rather than an overall loss of diversity.

Beta-diversity analyses more consistently identified differences in the overall microbial community structure between ADHD and controls, although this pattern was not observed in all studies. These findings suggest that changes between groups may capture ADHD-related microbial alterations more effectively than within-sample analysis.

### 4.2. Taxonomic Composition and Functional Interpretation

Despite heterogeneity across studies, several recurrent taxonomic patterns emerged. However, an interpretation of the findings should consider that a substantial proportion of included studies presented a moderate-to-high risk of bias, limiting the strength and certainty of the available evidence. A reduced abundance of *Faecalibacterium* and *Alistipes* was among the most consistent findings.

*Faecalibacterium* is a key producer of butyrate in the gut, playing a central role in the maintenance of intestinal barrier integrity, modulation of neuroinflammatory processes, regulation of microglial function, and neurotransmitter synthesis [45]. Furthermore, butyrate acts as an inhibitor of histone deacetylases (HDACs) [46], meaning that reduced butyrate availability may alter the expression of neurotrophic factors, such as brain-derived neurotrophic factor (BDNF) in the prefrontal cortex, a brain region critically involved in attention control and executive function, which is implicated in ADHD. Furthermore, *Faecalibacterium* was negatively correlated with ADHD symptom severity [24], possibly contributing to the hypothesis that it may play a protective role.

In contrast, *Alistipes* typically produces metabolites such as succinate and acetate and is involved in tryptophan metabolism [47]. Disruption of this metabolism can influence serotonin synthesis and the kynurenine pathway. Both have an important role in emotional regulation, the response to stress, and cognitive behavior [48]. On the other hand, several taxa increased. At higher taxonomic levels, the frequently reported increase in *Proteobacteria*, a phylum often enriched in pro-inflammatory pathobionts, is consistent with a low-grade inflammatory state and increased intestinal permeability, mechanisms proposed in ADHD pathophysiology [49]. Notably, *Odoribacter* is recognized as an important producer of butyrate and propionate, and it is considered a next-generation probiotic [50]. Findings regarding *Odoribacter* were inconsistent across studies. While one study reported increased abundance in ADHD populations [27], another observed reduced levels [39]. Discrepancies were also observed regarding other taxa such as *Bifidobacterium*, *Prevotella*, and *Parabacteroides*. These inconsistencies are likely driven by differences in the methodological approaches, age, dietary patterns, medication status, ADHD subtype, and comorbidities, as well as geographic and lifestyle factors. Therefore, the role of these taxa in ADHD remains uncertain and should be interpreted cautiously, highlighting the need for better phenotypic stratification in future studies regarding the microbiome in ADHD.

At the species level, *Ruminococcus gnavus* and *Bacteroides uniformis* were the most consistently increased. *Ruminococcus gnavus* has been associated with inflammatory bowel disease [51] and is known to be able to degrade intestinal mucin, thereby contributing to alterations in gut barrier functions [52]. In turn, *Bacteroides uniformis* is a commensal bacterium with immunomodulatory properties that produces metabolites such as succinate, acetate, and bile acids [53]. Interestingly, *Bacteroides uniformis* has been associated with beneficial effects on metabolic and immune function in other contexts [54]. However, its increased abundance in ADHD may reflect compensatory responses to dysbiosis or altered metabolic demands within the gut ecosystem.

The depletion of short-chain fatty acids (SCFAs) producers, such as *Faecalibacterium*, combined with an increase in mucin degraders such as *Ruminococcus gnavus* may compromise the intestinal barrier. This can allow pro-inflammatory and bacterial metabolites to cross into systemic circulation and ultimately reach the blood–brain barrier (BBB) [55].

These findings highlight that the taxonomic composition alone may not fully capture microbiome alterations associated with ADHD. Instead, the functional profiling of microbial metabolic pathways may provide more meaningful insights into gut–brain interactions. It is to be noted that these proposed biological pathways remain hypothetical because none of the included studies directly evaluated mechanistic gut–brain interactions.

### 4.3. Early-Life Microbiota

Longitudinal cohort studies provided particularly compelling findings, showing distinct microbial trajectories years before the ADHD diagnosis. These results strengthen the hypothesis that microbiota alterations are not merely a consequence of the disorder or its treatment, but could contribute to its developmental origins.

The early-life depletion of butyrate producers such as *Lachnospiraceae* and *Roseburia* and the reduction of 51 bacterial OTUs by six months may have significant neurodevelopmental consequences, possibly impairing the epigenetic regulation of neurotrophic factors like BDNF, which is a key mediator of synaptic plasticity and cognitive function [56]. Additionally, the increase in *Akkermansia muciniphila* during the first month of life [33], compared to its depletion in older cohorts [36], may suggest temporal shifts in the microbial composition. Although *Akkermansia muciniphila* is generally considered a beneficial commensal in adults [57], its increased abundance in early infancy could lead to excessive mucin degradation, potentially disrupting the immature intestinal barrier [58]. This suggests that the timing of microbial colonization may be as important as the identity of the taxa involved.

### 4.4. Intervention Effects

Intervention studies indicated that the gut microbiota is modifiable in individuals with ADHD. However, microbial changes were not always accompanied by clinical improvements nor were the symptom improvements always correlated with clear microbial alterations. This suggests that current interventions may not target the most relevant microbial pathways or that functionally meaningful changes may occur without detectable shifts in bacterial composition.

The success of early *Lactobacillus rhamnosus* GG supplementation [23] suggests that targeting the microbiota during the neonatal window may exert a prophylactic effect on neurodevelopmental trajectories. In contrast, interventions in established ADHD show more modest and localized effects. However, the influence of micronutrients on *Actinobacteria* and the impact of a low-lectin diet on *Lactobacillaceae* underscore the nutritional status as being a significant driver of microbial variance.

Psychostimulant medication was associated, in several studies, with distinct microbial profiles and lower alpha-diversity, raising the possibility that part of the microbiota signature attributed to ADHD may be related to medication. This should motivate more future research in treatment-naive children.

### 4.5. Microbiota and Clinical Outcomes

Several microbial taxa were reported to be correlated with ADHD symptom severity, particularly hyperactivity and emotional dysregulation. The negative association between *Faecalibacterium* and symptom scores is of particular interest, given its anti-inflammatory and butyrate-producing properties [59]. A key pathway underlying these clinical correlations may involve the vagus nerve, which provides a direct neural communication pathway between the gut and the brain [60]. SCFAs produced by beneficial bacteria act as signaling molecules that stimulate vagal afferents. These signals reach the locus coeruleus, which constitutes the brain’s primary hub for norepinephrine production [61]. Norepinephrine is essential for regulating arousal, attention, and executive function in the prefrontal cortex [61]. In ADHD, a microbiota-induced deficit in vagal stimulation may contribute to the understimulation of the prefrontal cortex, providing a biological explanation for why lower levels of SCFA-producing taxa correlate so strongly with increased inattention and hyperactivity. Additionally, these findings suggest that the microbial composition may influence the clinical expression of ADHD symptoms, rather than being only associated with the presence or absence of the disorder.

### 4.6. Insights from Mendelian Randomization

The results from Mendelian randomization analysis provide preliminary evidence for potential causal relationships between the gut microbiota composition and ADHD. The identification of specific taxa as risk-associated, such as the *Eubacterium hallii* group and *Ruminococcus torques* group, alongside protective taxa like *Butyricicoccus* and *Roseburia*, suggests that ADHD risk may be linked to the baseline gut microbial composition. In addition, the reverse causal signal observed for *Roseburia* is particularly illuminating. It suggests that ADHD-related behaviors, such as impulsivity and reward-seeking behavior, may drive dietary choices high in refined sugars and low in fiber. Such a diet actively inhibits the growth of beneficial and fiber-fermenting taxa like *Roseburia*, thereby reducing the production of neuroprotective SCFAs. This creates a self-perpetuating cycle where the behavioral manifestations of ADHD further degrade the gut environment, potentially exacerbating the severity of the disorder over time. Despite this study, findings remain inconsistent. Roseburia is suggested as a potential protective taxa in [35] but it is reported in other studies to be increased in the ADHD population [30,31]. Therefore, these findings may be interpreted with caution, requiring further investigation to better comprehend these alterations.

### 4.7. Mycobiome

Although still limited, the available evidence suggests that fungal communities may also contribute to ADHD-related gut–brain interactions. The observed increase in *Candida* species in individuals with ADHD along with developmental shifts in early-life fungal colonization, may point toward a broader ecological dysbiosis beyond bacterial communities. Importantly, fungal overgrowth often occurs in niches left behind by depleted bacterial communities [62]. *Candida* species can compromise the gut mucosal layer by promoting intestinal inflammation and disrupting epithelial tight junctions, acting synergistically with bacterial dysbiosis to increase intestinal permeability and systemic inflammation [62]. Fungal-related findings remain preliminary due to the limited number of available studies and should therefore be interpreted cautiously. Integrating the mycobiome into future ADHD research will likely be important to capture the full ecological complexity of the gut–brain axis.

### 4.8. Clinical and Research Implications

Taken together, the current evidence does not appear to support the existence of a single ADHD-specific microbial signature. Instead, ADHD appears to be associated with alterations in microbial pathways related to SCFA production, immune modulation, and neurotransmitter metabolism. These findings suggest that ADHD management may benefit from a stratified approach. The identification of early-life microbial trajectories may open a possible window for preventive approaches. This raises the possibility that, in the future, microbiome screening in infancy could help identify high-risk individuals who may benefit from targeted probiotics, like Lactobacillus rhamnosus GG, to stabilize neurodevelopmental paths.

For established ADHD, the findings emphasize that pharmacological treatment alone may be insufficient. In the longer term, adjunctive nutritional approaches may warrant investigation as complementary strategies, where diets and supplements are tailored to the individual’s specific microbial deficits (e.g., using prebiotics to boost *Faecalibacterium* in patients with high hyperactivity scores). Future studies should prioritize longitudinal designs beginning in early life, together with multi-omics approaches such as metagenomics and metabolomics. Greater methodological standardization, including consistent reporting of diversity metrics and improved control for confounding factors, as well as stratification by medication status and ADHD phenotype, will be crucial to improve comparability between studies. These approaches may help move the field beyond descriptive taxonomic findings and towards a clearer understanding of the metabolites and mechanisms underlying gut–brain interactions in ADHD. In fact, alterations in the gut microbiota may represent a potential area for future therapeutic investigation. However, current evidence remains insufficient to support clinical applications.

### 4.9. Strengths and Limitations of This Review

The primary strength of this systematic review lies in its integrative approach, synthesizing evidence from diverse study designs, including cross-sectional studies, longitudinal cohorts, randomized controlled trials, and Mendelian randomization analysis. By incorporating emerging evidence on the gut mycobiome and early-life trajectories, this work provides a more holistic view of the gut–brain axis than many previous reviews.

However, significant limitations remain, most notably the high degree of methodological heterogeneity (including small sample sizes, predominantly cross-sectional designs, differences in sequencing regions and in the reference databases used, inadequate control for key confounders such as diet, medication status, and probiotic use), which precluded the realization of a meta-analysis.

Additionally, most studies relied on 16S rRNA sequencing, thereby limiting our understanding to taxonomic shifts, leaving the actual functional and metabolic output of these communities largely unexplored. Furthermore, the inconsistent control for dietary patterns and medication usage across the included literature means that the ADHD-specific dysbiosis observed remains partially obscured by the noise of lifestyle and pharmacological confounders. This systematic review was not prospectively registered in PROSPERO, which may increase the risk of reporting bias.

## 5. Conclusions

This systematic review highlights a growing body of evidence connecting ADHD to changes in the gut microbiota composition. While no specific microbial signature related to ADHD has been identified, recurrent patterns suggests disruptions in microbial pathways involved in short-chain fatty acid production, immune modulation, intestinal barrier integrity, and neurotransmitter metabolism.

Specifically, the reduction of key butyrate-producing taxa alongside the enrichment of mucin-degrading and potentially pro-inflammatory microbes may contribute to mechanisms potentially linking gut dysbiosis with neurodevelopmental and behavioral outcomes.

Notably, longitudinal evidence indicates that microbiota alterations may emerge early in life, suggesting that the gut microbiome may contribute to the developmental origins of ADHD. More importantly, the timing of microbial colonization may be critical, suggesting that disruptions during early developmental windows may influence long-term gut-brain signaling.

While intervention studies demonstrate that the microbiota is modifiable, therapeutic approaches to date have shown variable clinical effects, suggesting that the most relevant microbial pathways have yet to be identified.

Overall, these findings suggest that ADHD is associated with functional disruptions within the gut–brain axis, rather than a single taxonomic signature. However, interpretation should remain cautious given the methodological heterogeneity and moderate-to-high risk of bias across the included studies. Future research should prioritize longitudinal cohort studies, multi-omics approaches, and improved methodological standardization to clarify the causal mechanisms and identify clinically meaningful microbial targets. Such advances may ultimately help determine whether microbiome-informed preventive strategies and personalized interventions for ADHD are feasible and clinically meaningful.

## Figures and Tables

**Table 1:** Characteristics of the included studies.

First Author, Year, and Country	Study Design	Population	Sample Size	Type of Intervention	No ADHD/No Healthy Controls	Sex (% Male)	Age, Mean [Range]	Method of ADHD Diagnosis	Time of Evaluation	Dietary Patterns	Probiotics and Antibiotics	ADHD Medication Status
Paarty, 2015, Finland [23]	Randomized double-blind, placebo-controlled prospective follow-up	Infants who had at least one family member with allergic disease were followed up to 13 years old	75	Probiotic treatment (perinatal supplementation Lactobacillus rhamnosus GG). During pregnancy: 1 × 10^10^ colony-forming units of Lactobacillus Rhamnosus GG or placebo (microcrystaline cellulose) daily for 4 weeks before expected delivery; after birth: 1x10^10 colony-forming units of Lactobacillus rhamnosus GG or placebo (microcrystaline cellulose) to the children up for 6 months daily (if breastfeeding, the supplementation was given to the mothers).	Placebo group: 5/30; Probiotic group: 0/40	Placebo group: 46% Probiotic group: 60%	[0–13]	ICD-10	3 weeks, 3, 6, 12, 18, 24 months, and 13 years	Not mentioned	Early probiotic supplementation was present in 58% of healthy children and 0% in children with ADHD. The use of antibiotics during the first 6 months of life was 20% and 33% in the placebo group and in the probiotic group, respectively. Two of the children with ADHD were on daily omega-3 fatty acid supplementation.	Mixed (4 children with ADHD were on methylphenidate)
Jiang, 2018, China [24]	Cross-sectional	Juvenile Chinese residents with ADHD and healthy controls	83	N/A	51/32	Control: 37.5% ADHD: 74.5%	Control: 8.5 ADHD: 8.47 [6–10]	K-SADS-PL scale based on DSM-IV	N/A	Excluded vegetarian and non-vegetarian diet (using questionnaire filled by parents)	No use of probiotic/prebiotic or antibiotics during 2 months prior to sample collection	Drug-naive
Prehn-Kristensen, 2018, Germany [25]	Cross-sectional	Male Caucasian children and adolescents with ADHD and healthy, born and raised in Germany	31	N/A	14/17	100%	Control: 13.1 DHD: 11.9	K-SADS-PL (Germany translation) based on DSM-IV-TR	N/A	The participants took notes on their food intake for fast food, meat/sausage/cold cuts, fruits/vegetables, and yoghurt/dairy products (using 4-point scales)	Not mentioned	Mixed (10 cases received methylphenidate. Nine of them discontinued the medication 48 h prior to samples collection)
Stevens, 2020, New Zealand [26]	Randomized controlled, double-blind trial	Children diagnosed with ADHD (13 New Zealand Europeans and 4 New Zealand Maori)	17	Micronutrient supplementation. Dose: capsules (formulation containing a blend of vitamins, minerals, amino acids, and antioxidants) or placebo to ADHD subjects	17/0	100%	Control: 9.3 Exposed: 10.29 [7–12]	Not mentioned	Baseline and weeks 2, 4, 6, 8 and 10	Dietary patterns, including consumption of fruit, vegetables, breakfast, and fast foods, were assessed at the baseline and end of the study with a higher score indicative of a healthier eating pattern	No use of antibiotics	Not mentioned
Wan, 2020, China [27]	Cross-sectional	Children with ADHD who were treated in the Pediatric Outpatient Department of the First Medical Center of the Chinese PLA General Hospital from January to June 2019 and healthy children	34	N/A	17/17	Control: 76.5% ADHD: 82.3%	Median Control: 8 ADHD: 8 [6–12]	K-SADS based on DSM-5	N/A	Excluded vegetarian diet. The participants were asked to maintain their regular dietary patterns for 1 week prior to sample collection (using food dairy)	No use of probiotics 1 month prior to sample collection	Not mentioned
Wang, 2020, Taiwan [28]	Cross-sectional	ADHD patients treated in the outpatient Department of Child Psychiatry at Chang Gung Children’s Hospital in Taiwan and healthy control children	60	N/A	30/30	Control: 60% ADHD: 76.7%	Control: 9.3 ADHD: 8.4 [6–16]	K-SADS-E and DSM-IV-TR	N/A	Excluded vegetarian diet (using FFQ). Diet of cases differed from that of controls	No use of probiotics or antibiotics	Drug-naive
Jung, 2022, South Korea [29]	Cross-sectional	Korean elementary school students	40	N/A	7/33	Information only for the whole sample: 65%	Information only for the whole sample: 11.825 [11–12]	K-ARS and the criteria of Lee	N/A	The dietary intake was analyzed using a dish-based semi-quantitative food frequency questionnaire. The dietary patterns of participants were divided into four groups: healthy, processed foods, fish/shellfish, and meat	No use of probiotics and antibiotics	Not mentioned
Lee, 2022, Taiwan [30]	Cross-sectional	Children with ADHD who were receiving no medication and a healthy control group of children without ADHD	76	N/A	54/22	Control: 59.1%; ADHD: 81.5%	Control: 9.73; ADHD: 8.39; [6–18]	DSM-5 and the Swanson, Nolan and Pelham Parent Rating Scale	N/A	Not mentioned	No use of probiotics in the 2 weeks prior to the sample collection	Drug-naive
Wang, 2022, Taiwan [31]	Cross-sectional	Children with ADHD from Chang Gung Children’s Hospital in Taiwan and healthy controls from the community close to the hospital	80	N/A	41/39	Control: 56.4% DHD: 73.2%	Control: 10 ADHD: 8 [6–16]	K-SADS-E and DSM-IV	N/A	Excluded vegetarian diet (using FFQ)	No use of probiotics and antibiotics 1 month prior to sample collection	Drug-naive
Bundgaard-Nielsen, 2023, Denmark [32]	Cross-sectional	Danish children and adolescents	95	N/A	32/31	Siblings: 57.1% Unrelated controls: 52.9% ADHD: 65.6%	Median Siblings: 8 Unrelated controls: 10 ADHD: 9 [5–17]	ICD-10, ADHD-rating scale, T.O.V.A	N/A	The parents of all participants were asked to describe the typical diet of the participating children	None of the participants received probiotic supplements. No use of antibiotics 3 months prior to sample collection	Mixed (46.9% of ADHD patients used ADHD medication: 73.3% used methylphenidate, 13.3% used Lisdexamfetamine, and 6.6% used atomoxetine)
Cassidy-Bushrow, 2023, USA [33]	Cohort	WHEALS birth cohort: Pregnant women aged 21–49 years, second trimester or later, with due dates from September 2003 through December 2007, living in the city of Detroit and the surrounding suburban areas	314	N/A	59/255	Control: 49.4% ADHD: 74.6%	At 10-year visit: Control: 9.96 ADHD: 9.97	Not mentioned	1 and 6 months	Not mentioned	Not mentioned	Not mentioned
Wang, 2023, Taiwan [34]	Cross-sectional	Children residents in Taiwan with ADHD and healthy controls	70	N/A	35/35	Control: 51.4% ADHD: 77.1%	Control: 10.2 ADHD: 8.2 [6–12]	DSM-5	N/A	Excluded vegetarian diet	Children included had not taken probiotics in the last 3 months and were not on antibiotic treatment	Drug-naive
Wang, 2023, China [35]	Two-sample mendelian randomization	GWAS (gene-wide association study) from people with European ancestry	216,593	N/A	830/215,763	Not mentioned	Not mentioned	ICD-10	N/A	N/A	N/A	N/A
Ahrens, 2024, Sweden [36]	Prospective longitudinal cohort	Children born in Sweden between 1997 and 1999 followed until early adulthood	16,640	N/A	933/14,869	Information only for the whole cohort 51.8%	No information for controls ADHD: 15.52	ICD-10, DSM-IV and DSM-5	N/A	Not mentioned	Those experiencing three or more penicillin-requiring infections during early childhood (birth to 5 years) were at high risk to develop ADHD (OR = 3.27 [2.29–4.67]). Children with at least six infections requiring antibiotics were more likely to develop ADHD (OR = 1.62–2.9)	Not mentioned
Kurokawa, 2024, Japan [37]	Cross-sectional	Japanese children clinically confirmed to have ADHD, their unaffected neurotypical siblings, and unrelated neurotypical controls	60	N/A	19/41 (13 neurotypical siblings and 28 unrelated neurotypical controls)	Unaffected patients siblings: 38% Non-related neurotypicals: 71.4% ADHD: 78.9%	Unaffected patients siblings: 8.2 Non-related neurotypicals: 8.1 ADHD: 7.7 [6–12]	Conners 3rd Edition DSM-5	N/A	At the time of enrollment, the Brief-type Diet History Questionnaire-15y (BDHQ-15y) was employed to collect basic information on the patients’ dietary habits. No significant differences found between groups	No use of antibiotics 3 months prior to sample collection	Mixed (in the ADHD group, 5 persons used atomoxetine, 5 persons used guanfacine, 2 persons used methylphenidate, and 1 person used aripiprazole)
Long, 2024, China [38]	Single-center prospective cohort	Children diagnosed with ADHD	58	Intervention group integrated a low-lectin diet	58/0	Control: 88.4% Experimental: 92.5%	Control: 10.4 Experimental: 10 [7–15]	DSM-5	Baseline (week 0) and endpoint (not mentioned)	The experimental group adhered to a low-lectin diet, while the control group maintained their original dietary habits	Not mentioned	Medicated (Both groups underwent standard medication treatment for ADHD)
Wang, 2024, China [39]	Cross-sectional	Children and adolescents, aged 6–16, that frequents Yan’na Third People’s Hospital and local schools	107	N/A	47/60	Control: 70% ADHD: 70.21%	Control: 9.88 ADHD: 9.28 [6–16]	ADHD rating scale-IV, based on DSM-5 criteria and K-SADS-PL	N/A	Not mentioned	No use of antibiotics, probiotics, and prebiotics for at least 1 month prior to the sample collection	Drug-naïve
Wang, 2024, Taiwan [40]	Randomized, double-blind, placebo-controlled clinical trial	Children with ADHD on stable methylphenidate treatment	76	Add-on Bifidobacterium bifidum (Bf-688; 5 × 10^9^ CFU/day) vs. placebo (maltodextrin + corn starch)	76/0	Probiotic group: 82.4% Placebo group: 88.2%	9.1 for both groups [6–12]	DSM-5	Baseline (week 0) and endpoint (week 12)	Patients who followed a vegetarian diet were excluded from the study. All participants were instructed not to make any alterations to their dietary habits	Patients who were taking probiotics or antibiotics were excluded from the study	Medicated (participants were on ongoing pharmacotherapy for ADHD (methylphenidate) at a stable dosage for at least 4 weeks prior)
Ast, 2025, North America (USA and Canada) [41]	Double-blind randomized controlled trial	Children with ADHD from the MADDY study	44	Participants received micronutrients or a placebo for 16 weeks. The 36-ingredient micronutrients contained all known vitamins and essential minerals, while the placebo contained cellulose and riboflavin	44/0	Placebo group: 63.6% Micronutrient group: 72.7%	Placebo: 9.4; Intervention: 9.7 [6–12]	DSM-5	Baseline, week 8 (end RCT) and week 16 (open extension)	Not mentioned	Participants were asked to discontinue probiotics if reported at the initial visit. Antibiotic history was recorded with no restrictions on participation for recent antibiotic use	Drug-free (participants had to be psychotropic medication-free for at least 2 weeks before the enrollment visit)
Boonchooduang, 2025, Thailand [42]	Cross-sectional	Children with ADHD and healthy controls	30	N/A	20/10	90%	Control: 9.56; ADHD (medicated): 9.50; ADHD non-medicated: 8.93 [6–12]	DSM-5	N/A	Dietary intake assessed via food frequency questionnaires, no significant differences between ADHD and heathy controls	Participants were excluded if use of probiotics/prebiotics or antibiotics in the previous 14 days	Mixed (50% of the ADHD group was on ADHD medication (methylphenidate))
Novau-Ferre, 2025, Spain [43]	Randomized, double-blind, placebo-controlled trial	Children aged 5–14 years with ADHD or ASD from Tarragona (Spain)	39	Probiotic supplementation vs. placebo for 12 weeks	39/0	71.8%	10 [7–12]	DSM-5	12 weeks	Information on food intake was obtained using a validated 45-item semi-quantitative food consumption frequency questionnaire	Children included had not taken probiotics in the last 3 months and were not on antibiotic treatment	Mixed (ADHD medication used by 9 children)
Trezzi, 2025, Italy [44]	Randomized, double-blind, comparison-controlled clinical trial	Drug-naïve school-aged children diagnosed with ADHD	38	Supplementation with an experimental symbiotic mix enriched with pigmented corn extract (EXP) compared with a symbiotic mix of probiotics and prebiotic fibers (COMP)	38/0	EXP: 70% COMP: 90.5%	EXP: 11.95 COMP: 12.19 [6–16]	DSM-5	Baseline (T0) and after 3 months of supplementation (T3)	Not mentioned	Participants were randomly assigned to receive either a synbiotic mix of probiotics plus prebiotic fibers or the experimental synbiotic mix composed of the same probiotics and prebiotic fiber, with the additional presence of a vegetable extract derived from an Italian pigmented corn variety, rich in anthocyanin	Drug-naïve

**Abbreviations:** ADHD, attention-deficit/hyperactivity disorder; ASD, autism spectrum disorder; CFU, colony-forming units; DSM-5, Diagnostic and Statistical Manual of Mental Disorders, Fifth Edition; DSM-IV, Diagnostic and Statistical Manual of Mental Disorders, Fourth Edition; DSM-IV-TR, Diagnostic and Statistical Manual of Mental Disorders, Fourth Edition, Text Revision; FFQ, Food Frequency Questionnaire; ICD-10, International Classification of Diseases, 10th Revision; K-ARS, Korean ADHD Rating Scale; K-SADS-PL, Kiddie Schedule for Affective Disorders and Schizophrenia for School-Age Children—Present and Lifetime Version; K-SADS-E, Kiddie Schedule for Affective Disorders and Schizophrenia—Epidemiologic Version; N/A, not applicable; OR, odds ratio; RCT, randomized controlled trial; T.O.V.A., Test of Variables of Attention.

**Table 2:** Microbiota/microbiome sampling and analytical methodology.

Study	Sample Storage	DNA Extraction Method	Sequencing Method	Target	Database
**[23]**	Stored at −80 °C until analyzed	Automated KingFisher DNA extraction System and InviMag Stool DNA kit	Not mentioned	Not mentioned	Not mentioned
**[24]**	Stored at −80 °C within 30 min	QIAamp DNA Stool Mini kit	Illumina MiSeq	16S rRNA (V3–V4 region)	Not mentioned
**[25]**	Collected in Sarstedt fecal collection tubes and stored at 4 °C until preparation. Extracted DNA was stored at −80 °C	FastDNA Spin kit for Soil	Illumina MiSeq	16S rDNA (V1–V2 region)	Not mentioned
**[26]**	Stored at −4 °C for a maximum of 14 days, before aliquoting and storage at −80 °C	NucleoSpin DNA Stool Isolation kit	Illumina MiSeq	16S rRNA (V3–V4 regions)	GreenGenes
**[27]**	Stored in a sterile plastic cup at −80 °C prior to testing	HiPure Stool DNA kits	Shotgun metagenomic sequencing (Illumina NovaSeq)	Whole genome	MetaPhlAn2
**[28]**	Stored at −4 °C immediately after collection and then at −80 °C within 24h	QIAamp DNA Stool Mini kit	Illumina MiSeq	16S rRNA (V3–V4 region)	RDP
**[29]**	Not mentioned	DNeasy Blood and Tissue Kit	emPCR Amplification 7020 Thermal cycler	16S rRNA (V3–V4 regions)	EzBioCloud
**[30]**	Not mentioned	QIAamp PowerFecal DNA kit	Illumina MiSeq	16S rRNA (V3–V4 region)	SILVA, GreenGenes
**[31]**	Stored at 4 °C after collection and then at −80 °C within 24h	QIAamp PowerFecal DNA Kit from Qiagen	Illumina MiSeq	16S rRNA (V3–V4 region)	SILVA
**[32]**	Stored at −80 °C	QIAamp PowerFecal DNA Kit automated on a QIAcube	Illumina MiSeq	16S rRNA (V4 region)	SILVA
**[33]**	Stored at −80 °C	CTAB buffer-based protocol	Illumina NextSeq (bacterial sequencing) and Illumina MiSeq (fungal sequencing)	Bacterial: 16S rRNA (V4 region) Fungal: ITS 2 of the rRNA	GreenGenes
**[34]**	Not mentioned	QIAamp DNA Stool Mini Kit	Illumina Miseq	ITS86F and ITS4R	RDP
**[35]**	N/A	N/A	Not mentioned	16S rRNA (V4, V3–V4 and V1–V2 regions)	Not mentioned
**[36]**	Stored at −80 °C until analyzed	Not mentioned	Illumina MiSeq	16S rRNA (V3–V4 regions)	SILVA
**[37]**	Stored in a −18 °C household freezer immediately after collection. It was sent within 48 h using a frozen courier service at −15 °C. In the laboratory, samples were stored at −80 °C until analysis	Lyophilization followed by extraction with zirconia beads and phenol/chloroform/isoamyl alcohol method	Illumina MiSeq	16S rRNA (V1–V2 region)	SILVA
**[38]**	Not mentioned	Not mentioned	Not mentioned	16S rDNA sequencing	Not mentioned
**[39]**	Stored at −80 °C	QIAamp PowerFecal pro DNA Kit	Metagenomic shotgun sequencing	Whole genome	UHGG
**[40]**	Not mentioned	QIAamp Fast DNA Stool Mini Kit	Illumina MiSeq	16S rRNA (V3–V4 regions)	GreenGenes
**[41]**	The sample was stored at room temperature and was returned at the next visit (within days of collection) and immediately stored at −80 °C until analysis	Quick-DNA Fecal/Soil Microbe Miniprep Kit (Zymo)	Illumina MiSeq	16S rRNA (V4 region)	SILVA
**[42]**	Refrigerated immediately at −4 °C and stored at −80 °C within 24 h until analysis.	QIAamp PowerFecal Pro DNA kit	Illumina MiSeq	16S rRNA (V3–V4 regions)	SILVA
**[43]**	Stored at −20 °C until further processing	QIAamp PowerFecal Pro DNA kit	Ion Torrent sequencing	16S rRNA (V4 region)	SILVA
**[44]**	Stored at −80 °C	QIAamp Power Fecal Pro DNA kit	Illumina NovaSeq	16S rRNA (V3–V4 regions)	SILVA

**Abbreviations:** CTAB, cetyltrimethylammonium bromide; DNA, deoxyribonucleic acid; emPCR, emulsion polymerase chain reaction; ITS, internal transcribed spacer; RDP, Ribosomal Database Project; rDNA, ribosomal deoxyribonucleic acid; rRNA, ribosomal ribonucleic acid; UHGG, Unified Human Gastrointestinal Genome.

**Table 3:** Microbial diversity and microbial profile in each study.

Study	Alpha-Diversity	Findings	Beta-Diversity	Findings	Taxonomic Findings	Microbiota and ADHD Symptoms
**Cross-sectional studies (ADHD vs. healthy controls)**						
**[24]**	Chao1, Shannon, Simpson	No significant differences	Bray–Curtis and UniFrac (weighted and unweighted)	No significant differences	⭡ *Peptostreptococcaceae*, *Moraxellaceae*, *Xanthomonadaceae*, *Peptococcaceae* ⭣ *Alcaligenaceae*, *Faecalibacterium*, *Lachnoclostridium*, *Dialister*, *Sutterella*	The abundance of *Faecalibacterium* was negatively associated with the total Conners Parent Rating Scales (CPRS) score. *Faecalibacterium* was also negatively associated with the hyperactivity index score
**[25]**	Observed species, Chao1, and Shannon	⭣ Shannon in ADHD vs. controls Mothers of ADHD patients also had a reduction in alpha-diversity	Bray–Curtis	Significant differences	⭡ *Neisseriaceae*, *Bacteroidaceae*, *Neisseria*, *Bacteroides* ⭣ *Prevotellaceae*, *Catabacteriaceae*, *Porphyromonadaceae*, *Prevotella*, *Parabacteroides*	*Bacteroides* spp. correlated with levels of hyperactivity and impulsivity
**[27]**	Chao1, Shannon, and Simpson	No significant differences	Euclidean distance	No significant differences	⭡ *Odoribacteraceae*, *Enterococcaceae*, *Odoribacter*, *Enterococcus*, *Bacteroides caccae*, *Odoribacter splanchnicus*, *Paraprevotella xylaniphila*, *Veillonella parvula* ⭣ *Ruminococcaceae*, *Veillonellaceae*, *Faecalibacterium*, *Faecalibacterium prausnitzii*, *Lachnospiraceae bacterium*, *Ruminococcus gnavus*	Not investigated
**[28]**	Observed OTUs, Chao1, Shannon, Simpson, and ACE indices	⭡ Chao1 and Shannon in ADHD vs. controls ⭣ Simspon in ADHD vs. controls	UniFrac (weighted and unweighted)	No significant differences between groups	⭡ *Fusobacteria*, *Fusobacterium*, *Bacteroides uniformis*, *Bacteroides ovatus*, *Sutterella stercoricanis* ⭣ *Lactobacillus*, *Bacteroides coprocola*	*Sutterella stercoricanis* and *Bacteroides ovatus* had a positive correlation with ADHD symptoms (SNAP-IV teacher and parent forms)
**[29]**	Not mentioned	Alpha-diversity of the ADHD group was markedly lower compared to the control group	Not mentioned	Heat map analysis showed that the microbiota profiles of the control and ADHD groups mostly belonged to distinct clusters	⭡ *Escherichia*, *Enterobacter*, *Clostridium* ⭣ *Bifidobacterium*, *Faecalibacterium*, *Ruminococcus*	Correlation analysis showed a positive correlation between ADHD scores and *Escherichia* strains and a negative correlation between ADHD scores and *Bifidobacterium*, *Ruminococcus*, and *Faecalibacterium* strains
**[30]**	Chao1, Shannon, Simpson, and ACE indices	No significant differences	UniFrac (weighted and unweighted) distances	Significantlydecrease in children with ADHD vs. controls	⭡ *Proteobacteria*, *Betaproteobacteriales*, *Burkholderiaceae*, *Acidaminococcaceae*, *Agathobacter*, *Phascolarctobacterium*, *Prevotella*, *Acidaminococcus*, *Roseburia*, *Parasutterella*, *Ruminococcus gnavus* ⭣ *Rikenellaceae*, *Alistipes*, *Eubacterium eligens*	No significant correlation was identified between bacteria and core ADHD symptoms using SNAP-IV scores. However, *Ruminococcus gnavus* group was associated with rule-breaking and aggressive behavior. *Acidaminococcus* was associated with thought problems
**[31]**	Observed ASV, Shannon, Simpson, and ACE indices	No significant differences	UniFrac (weighted and unweighted) distances	No significant differences	⭡ *Ruminococcaceae*, *Lachnospiraceae*, *Agathobacter*, *Anaerostipes*, *Roseburia*, *Bacteroides uniformis*, *Firmicutes/Bacteroides* ratio	Lower plasma TNF-α levels in ADHD patients were negatively correlated with both gut microbiome diversity and the severity of ADHD symptoms
**[32]**	ASV richness, Shannon, and Faith’s PD indices	⭣ ASV richness and Faith’s PD in ADHD vs. non-affected children	UniFrac (weighted and unweighted) and Bray-Curtis distances	Significant differences between the ADHD group and healthy controls	⭡ *Streptococcus*, *Lactobacillus*, *Hungatella*, *Eggerthella*, *Ruminococcus gnavus* ⭣ *Coprobacter*, *Bilophila*, *Howardella*, *Colidextribacter*, *Alistipes*	Not investigated
**[34]**	Observed species, Chao1, and Shannon indices	No significant differences	Bray–Curtis distance	Significant differences between groups	⭡ *Ascomycota*, *Candida albicans*, *Candida tropicalis* ⭣ *Basidiomycota*	Not investigated
**[37]**	Chao1, Shannon, and Faith’s phylogenetic diversity indices	No significant differences	UniFrac (weighted and unweighted) distances	No significant differences	Not investigated	Not investigated
**[39]**	Chao1, Shannon, Simpson indices	⭣ Shannon and Simpson indices in the ADHD group	Bray–Curtis, Euclidean, and JSD distances	⭡ in ADHD group	⭡ *Anaerostipes hadrus*, *Lachnospira*, *Phascolarctobacterium faecium* ⭣ *Bacteroides*, *Odoribacter*, *Alistipes*, *Megamonas*, *Bacteroides caccae*, *Odoribacter splanchnicus*	Not investigated
**[42]**	Observed species, Shannon, Pielou’s evenness, and Faith’s phylogenetic diversity	Unmedicated ADHD vs. healthy controls: ⭡ Faith’s PD in unmedicated ADHD Medicated ADHD vs. unmedicated ADHD: ⭣ Pielou’s evenness, Shannon index, and Faith’s PD in medicated ADHD	Jaccard and UniFrac (weighted and unweighted)	Significant differences between groups	Unmedicated ADHD vs. controls: ⭣ *Tyzzerella*, *Prevotellaceae NK3B31 group*, *Blautia*, *Anaerostipes*, *Verrucomicrobiota*, *Desulfobacterota*, *Bacteroidota*, *Firmicutes*, *Actinobacteriota*. ⭡ *Proteobacteria* Medicated ADHD vs. unmedicated ADHD: ⭣ *Ruminococcaceae*, *Haemophilus*, *Kytococcus*, *Micrococcus*, *Staphylococcus*, *Corynebacterium*, *Brevundimonas*, *Odoribacter*. ⭡ *Anaerostipes*	*Tyzzerella* is negatively associated with inattention symptom severity
**RCT’s (pre-post intervention or placebo vs. probiotic)**						
**[23]**	Not investigated	N/A	Not investigated	N/A	Specific taxa in infancy were associated with later ADHD outcomes: ⭣ *Bifidobacterium*, *Bifidobacterium longum*, *Bacteroides*, *Lactobacillus-Enterococcus* group, *Clostridium histolyticum*	Early probiotic supplementation with *Lactobacillus rhamnosus* GG was associated with a reduced risk of development of ADHD later in childhood
**[26]**	Observed OTUs, Shannon, Faith’s PD	A significant difference in the change in observed OTUs was found between the treatment and placebo groups. However, within each group there was no significant change	UniFrac (weighted and unweighted) distances	No significant differences	After intervention: ⭡ *Coriobacteriales*, *Collinesella aerofaciens*, *Proteobacteria* ⭣ *Actinobacteria*, *Bifidobacteriales*, *Bifidobacterium*	A higher relative abundance of *Bifidobacterium* and a decrease of *Actinobacteria* was associated with a lower ADHD score
**[40]**	Chao1 and Shannon indices	Shannon ⭡ in both groups (pre-post)–NS between groups Chao1 ⭡ in both groups (pre-post)–NS between groups	UniFrac (weighted and unweighted) distances	Significant variation	⭡ *Firmicutes/Bacteroidetes* ratio (probiotic only)	Scores related to inattention and hyperactivity registered significant reductions in both groups. However, the probiotic group outperformed the placebo group in terms of reduced omission errors and improved response rates in visual and auditory attention
**[41]**	Observed taxa, Shannon, Inverse Simpson, and Pielou evenness indices	⭡ Simpson index and Pielou’s evenness in micronutrient ⭣ Simpson index and Pielou’s evenness in the placebo	Bray–Curtis	Significantly different in the micronutrients group vs. placebo	⭡ *Rikenellaceae*, *Oscillospiraceae* ⭣ *Actinobacteriota*, *Bifidobacteriaceae* in micronutrient vs. placebo	In those children with a positive behavioral response, an increase was noted in the families *Oscillospiraceae* and *Rikenellaceae*. The reduction in *Actinobacteriota* was not linked with symptom improvement
**[43]**	Chao1, Shannon, Inverse Simpson, and Fisher’s alpha indices	No significant changes after probiotic supplementation	Bray–Curtis	No significant differences following probiotic intervention	Effect of probiotics in ADHD: ⭡ *Odoribacter* ⭣ *Eggerthellaceae*, *Escherichia*-*Shigella*	Not investigated
**[44]**	N/A	N/A	N/A	N/A	Between groups: Bifidobacterium in COMP at T0 (not observed at T3) Within groups: *Agathobacter* in both COMP and EXP, *Lachnospiraceae* in EXP, *Bacteroides* and *Faecalibacterium* in COMP, *Clostridium sensu stricto* in EXP	Parental ratings of ADHD symptoms showed small improvements over time in both groups of participants, not suggesting any superiority between the formulas
**Cohort**						
**[33]**	Shannon, Pielou’s evenness, Faith’s PD indices	⭡ Faith’s PD in children with ADHD at 6 months of age	UniFrac (weighted and unweighted) distances, Bray-Curtis	No significant differences in β-diversity between children who later developed ADHD and those who did not	Specific taxa in infancy were associated with later ADHD outcomes: ⭡ *Collinsella*, *Akkermansia*, *Dorea*, *Blautia* ⭣ *Lachnospiraceae*, *Enterococcus*, *Ruminococcus*, *Lactobacillales*	Not investigated
**[36]**	Not investigated	N/A	Not investigated	N/A	Specific taxa in infancy were associated with later ADHD outcomes: ⭡ *Bacteroides*, *Megamonas funiformis* ⭣ *Akkermansia muciniphila*, *Phascolarbacterium faecium*, *Roseburia hominis*, *Coprococcus eutactus*, *Alistipes putredinis*, *Bacteroides ovatus*	Inverse correlation with mood symptoms: *Akkermansia*, *Coprococcus*, *Roseburia*
**[38]**	Observed OTUs, Chao1, Shannon, and Simpson indices	No significant differences	Euclidean distance	Revealed significant grouping, indicating that dissimilarities between groups were greater than within groups	After intervention: ⭡ *Lactobacillaceae*, *Pasteurellales*, *Pastereullaceae*	Significant improvement in ADHD symptom scores following the low-lectin diet. However, there were no significant differences between the experimental and control group post-treatment
**Mendelian randomization**						
**[35]**	N/A	N/A	N/A	N/A	Correlated with risk of developing ADHD: ⭡ *Eubacterium hallii* group, *Ruminococcaceae* ⭣ *Butyricicoccus*, *Roseburia*, *Desulfovibrio*, *Lachnospiraceae*, *Romboutsia*, *Oxalobacteraceae*, *Peptostreptococcaceae*	Not investigated

**Abbreviations**: ACE, abundance-based coverage estimator; ADHD, attention-deficit/hyperactivity disorder; ASV, amplicon sequence variant; CPRS, Conners’ Parent Rating Scale; JSD, Jensen–Shannon divergence; N/A, not applicable; NS, not significant; OTUs, operational taxonomic units; PD, phylogenetic diversity; SNAP-IV, Swanson, Nolan, and Pelham Questionnaire IV; T0, baseline; T3, 3 months; TNF-α, tumor necrosis factor alpha; ⭡, increased; ⭣, decreased.

**Table 4:** Summary of the results from the included studies regarding ADHD vs. controls signature. Taxa reported in fewer than two studies were not included in the evidence map to improve interpretability. Only cross-sectional studies were included in this heatmap.

	Alpha-Diversity						Beta-Diversity					Phylum			Family				Genera											Species			
	Observed Taxa	Chao1 index	Shannon index	Simpson index	Faith’s PD index	Pielou evenness index	Unweighted UniFrac distance	Weighted UniFrac distance	Bray-Curtis dissimilarity	Euclidean distance	Jaccard distance	*Actinobacteria*	*Firmicutes*	*Proteobacteria*	*Rikenellaceae*	*Porphyromonadaceae*	*Prevotellaceae*	*Ruminococcaceae*	*Agathobacter*	*Roseburia*	*Alistipes*	*Anaerostipes*	*Bifidobacterium*	*Eggerthella*	*Odoribacter*	*Faecalibacterium*	*Parabacteroides*	*Lactobacillus*	*Prevotella*	*Ruminococcus gnavus*	*Bacteroides caccae*	*Odoribacter splanchnicus*	*Bacteroides uniformis*
24																																	
25																																	
27																																	
28																																	
29																																	
30																																	
31																																	
32																																	
34																																	
37																																	
39																																	
42																																	
																																	
									Increased																								
									Decreased																								
									Significant differences																								
									No differences																								
									Not mentioned																								

## Data Availability

This systematic review has been retrospectively registered in the Open Science Framework (OSF). Data are available on reasonable request.

## Supplementary Materials

The following supporting information can be downloaded at: https://www.mdpi.com/article/10.3390/microorganisms14061301/s1, Table S1: PRISMA 2020 Checklist; Table S2: Summary of risk of bias assessment of the included studies; Table S3: Domain-level judgments in RoB 2 included studies.

## Abbreviations

The following abbreviations are used in this manuscript:

ACE	Abundance-based Coverage Estimator
ADHD	Attention-Deficit/Hyperactivity Disorder
ADHD-IV-RS	ADHD Rating Scale IV
ASV	Amplicon Sequence Variant
BBB	Blood-Brain Barrier
BDNF	Brain-Derived Neurotrophic Factor
Bf-688	*Bifidobacterium bifidum* strain Bf-688
CBCL	Child Behaviour Checklist
C-GAS	Child Global Assessment Scale
CNS	Central Nervous System
CPT	Continuous Performance Test
DNA	Deoxyribonucleic Acid
DSM-IV	Diagnostic and Statistical Manual of Mental Disorders, Fourth Edition
DSM-IV-TR	Diagnostic and Statistical Manual of Mental Disorders, Fourth Edition, Text Revision
DSM-5	Diagnostic and Statistical Manual of Mental Disorders, Fifth Edition
FFQ	Food Frequency Questionnaire
GABA	Gamma-Aminobutyric Acid
GWAS	Genome-Wide Association Studies
HDACs	Histone Deacetylases
ICD-10	International Classification of Diseases, 10th Revision
ITS	Internal Transcribed Spacer
JBI	Joanna Briggs Institute
K-ARS	Korean ADHD Rating Scale
K-SADS	Kiddie Schedule for Affective Disorders and Schizophrenia
MR	Mendelian Randomization
MeSH	Medical Subject Headings
OTUs	Operational Taxonomic Units
PRISMA	Preferred Reporting Items for Systematic Reviews and Meta-Analyses
RDP	Ribosomal Database Project
RoB	Risk of Bias
RoB 2	Revised Cochrane Risk of Bias tool for randomized trials
ROBINS-I	Risk of Bias In Non-randomized Studies–of Interventions
ROB-MR	Risk of Bias in Mendelian Randomization
RCT	Randomized Controlled Trial
SCFAs	Short-Chain Fatty Acids
SDQ	Strengths and Difficulties Questionnaire
SNAP-IV	Swanson, Nolan and Pelham Questionnaire IV
UHGG	Unified Human Gastrointestinal Genome
